# Identification of cancer risk groups through multi-omics integration using autoencoder and tensor analysis

**DOI:** 10.1038/s41598-024-59670-8

**Published:** 2024-05-17

**Authors:** Ali Braytee, Sam He, Shuxian Tang, Yuxuan Sun, Xiaoying Jiang, Xuanding Yu, Inder Khatri, Kunal Chaturvedi, Mukesh Prasad, Ali Anaissi

**Affiliations:** 1 School of Computer Science, University of Technology Sydney, Ultimo, 2007 Australia; 2 School of Computer Science, The University of Sydney, Camperdown, 2006 Australia; 3 TD School, University of Technology Sydney, Ultimo, 2007 Australia; 4 Department of Applied Mathematics, Delhi Technological University, Delhi, 110042 India

**Keywords:** Breast cancer, Cancer genomics, Cancer prevention, Computational models, Data mining, Machine learning, Predictive medicine

## Abstract

Identifying cancer risk groups by multi-omics has attracted researchers in their quest to find biomarkers from diverse risk-related omics. Stratifying the patients into cancer risk groups using genomics is essential for clinicians for pre-prevention treatment to improve the survival time for patients and identify the appropriate therapy strategies. This study proposes a multi-omics framework that can extract the features from various omics simultaneously. The framework employs autoencoders to learn the non-linear representation of the data and applies tensor analysis for feature learning. Further, the clustering method is used to stratify the patients into multiple cancer risk groups. Several omics were included in the experiments, namely methylation, somatic copy-number variation (SCNV), micro RNA (miRNA) and RNA sequencing (RNAseq) from two cancer types, including Glioma and Breast Invasive Carcinoma from the TCGA dataset. The results of this study are promising, as evidenced by the survival analysis and classification models, which outperformed the state-of-the-art. The patients can be significantly (*p*-value<0.05) divided into risk groups using extracted latent variables from the fused multi-omics data. The pipeline is open source to help researchers and clinicians identify the patients’ risk groups using genomics.

## Introduction

The subdivision of cancer and identifying risk groups are significant in medicine for diagnosing and treating cancer. Currently, in clinical practice, cancers are commonly treated according to their histological origin and pathological features. This approach has some limitations, such as similar histopathological features in some tumour masses, but their clinical presentation is quite different and corresponds to other risk groups. Several studies^1–4^ have shown that the pathological system of tumours at the molecular level is well characterised in terms of their parthenogenesis and stage of development. Fortunately, as the Human Genome Project progresses and new sequencing technologies continue to emerge and spread, a wealth of omics data is being generated that contributes to a better understanding of the issues involved. Nevertheless, due to the inherent complexity of biological systems, there is a limit to the information provided by a single piece of omics data. Genomic variation caused by somatic mutations, epigenetic changes, individual differences and environmental influences is possible during tumour development. The traditional analyses based on individual omics cannot capture the heterogeneity of all biological processes ^5^. On the other hand, using omics data also poses statistical modelling and computational challenges. In some omics data, there is the problem of a small number of samples and a large number of features ^6^. Therefore, these problems with single-omics hinder the better identification of risk groups or clinical phenotypes. Recently, there has been a growing trend towards studying and analyzing multi-omics data, including genomics, epigenomics, transcriptomics, proteomics, metabolomics, microbiomics, imaging, and others. The use of integrated data analysis has various advantages. It compensates for the lack of information in single-omics data and provides an integrated view of cancer analysis at the molecular level. This approach can play an essential role in assessing metastasis and selecting treatments for patients, thus contributing to the development of precision medicine. Few studies have used autoencoders in deep learning to extract features of multi-omics data and use these new features to build predictive models  ^5,7^. Furthermore, some studies have used unsupervised feature extraction for multi-omics based on tensor decomposition ^8,9^. However, the small omics datasets have not been considered, and the identification of risk groups from multiple omics data has not been investigated. Furthermore, combining multi-omics to generate a large matrix can cause the loss of information from smaller-sized omics. Information may be missing when the feature extraction or selection methods are implemented on this large integrated matrix ^10^.

**Figure 1:**
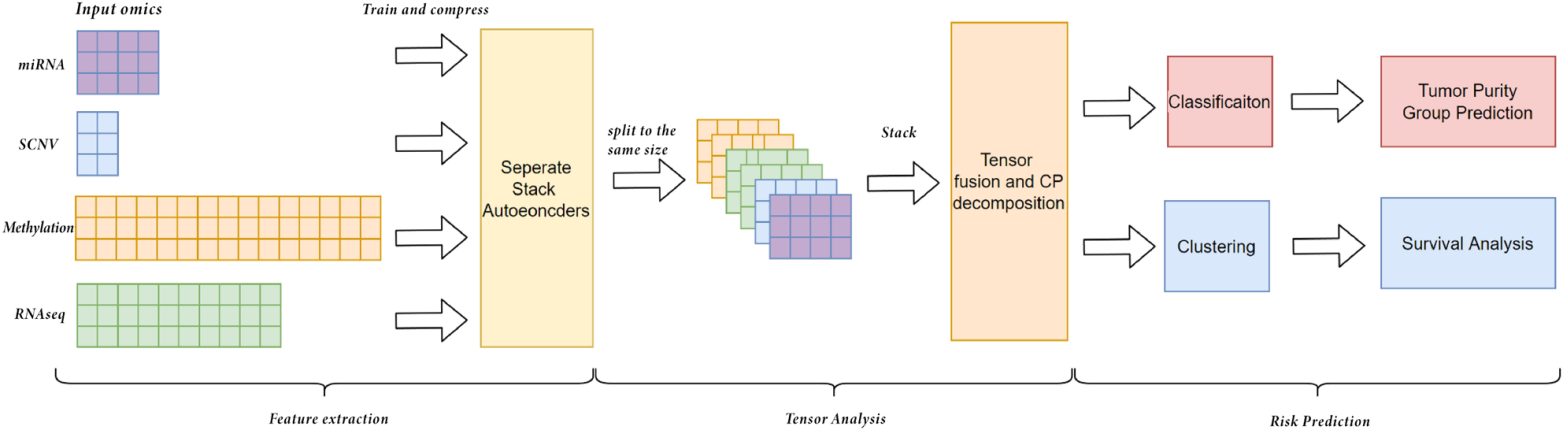
Our proposed framework contains three main components: feature extraction, tensor analysis, and risk prediction.

In this work, we developed a multi-omics feature learning framework as depicted in Fig. 1 to stratify patients into high-risk and low-risk groups by minimising information loss and learning significant features. Autoencoders are used as a dimensionality reduction method to capture the non-linear relationships between the data to maximise the retention of the original information in each single-omics data. Then, the latent variables of each omic are concatenated, and further feature learning is carried out using tensor analysis. Combining deep learning and tensor analysis avoids overweighting omics datasets due to high dimensionality while learning important common features across multi-omics.

The practical relevance of the results generated by the proposed framework is evident. Specific risk groups could be detected earlier based on the framework results, which help clinicians choose more appropriate therapies at different stages of treatment. Meanwhile, tensor analysis of multi-omics combined with deep learning methods may inspire more ways to identify cancer risk groups from the molecular level. Our contributions are summarized as follows:

- We propose a non-linear multi-omics method that considers the non-linear relationships between features in the assays.
- We integrate Tensors in the proposed model to extract expressive feature sets that capture important patterns and relationships in the data.
- We thoroughly evaluate our methods on two public datasets: Glioma and Breast Invasive Carcinoma. Our results are highly promising, as the survival analysis and classification models indicate.

## Results

The evaluated datasets are downloaded from the public LinkedOmics repository (http://linkedomics.org) including four single-omics datasets of SCNV, methylation, miRNA and RNAseq in addition to the clinical dataset for each cancer type. Breast Invasive Carcinoma and Glioma are the only two types with more than 600 clinical samples compared with all other cancer types. The chosen four single-omics datasets also have sample sizes of over 600, which are sufficient for the analysis. The core consistency diagnostic technique (CORCONDIA) suggests the rank $R=9$ for the Breast multi-omics tensor and 5 for Glioma tensor^11^. The mean square error for CP Decomposition is notably small for both the Glioma and Breast datasets, measuring 0.013 and 0.015, respectively. This underscores the minimal discrepancy between the original data and its decomposed form. We also compared our proposed method to MOFA^12^.

### Understanding biomarkers impact on latent variables

To investigate the contribution of biomarkers to the latent variables of omics data, we utilized the SHAP (SHapley Additive exPlanations) technique. This method is commonly used in explainable artificial intelligence to understand how a model makes predictions. The magnitude and directionality of SHAP values provide insights into each biomarker’s role in shaping the autoencoder’s latent variables.

#### Biomarkers impact using SHAP for glioma

**Figure 2:**
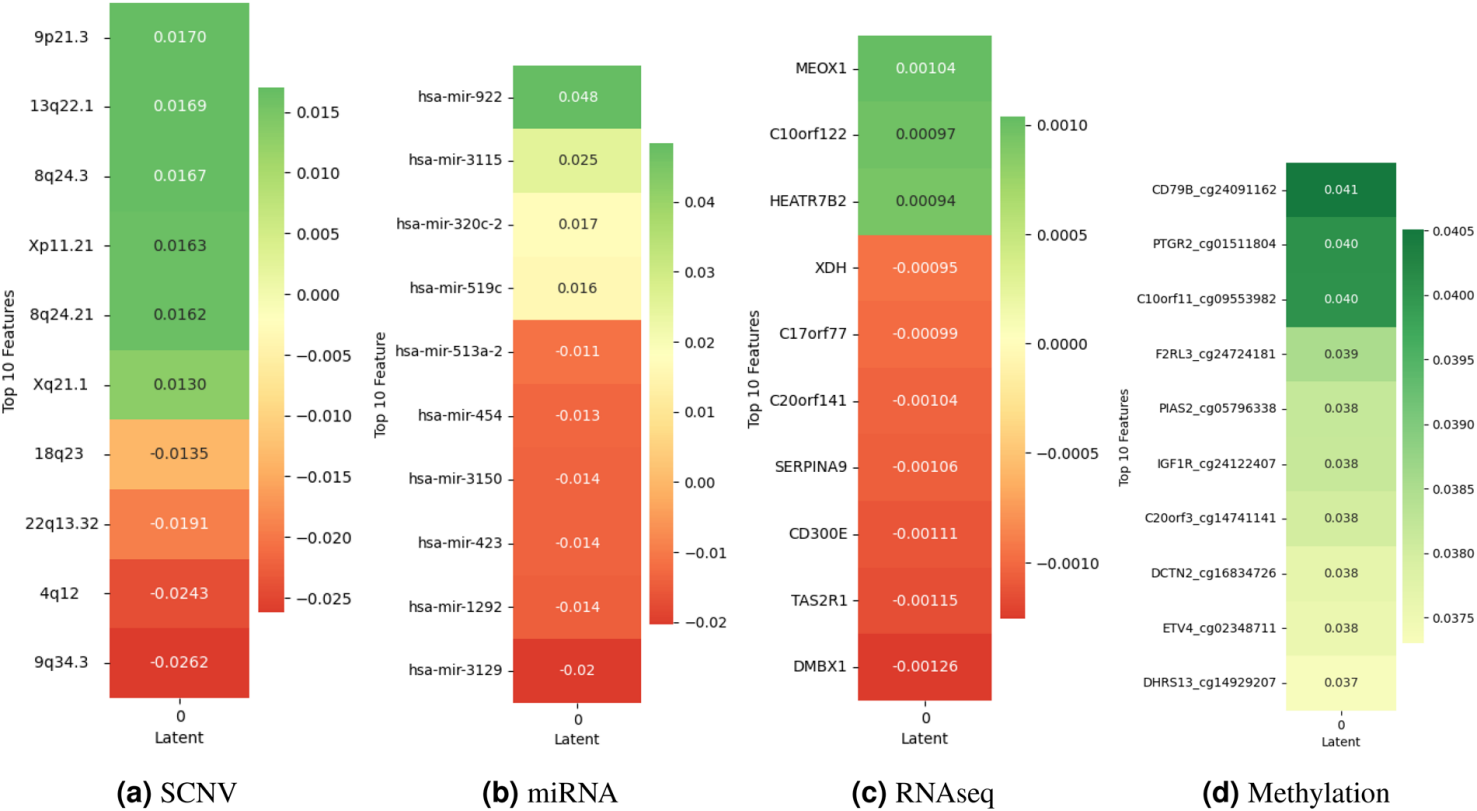
SHAP values demonstrate the impact of biomarkers on contributing to the latent variables of the autoencoder in Glioma multi-omics data.

In Fig. 2, we identified the top 10 crucial biomarkers within the Glioma dataset based on SHAP values. The SHAP values highlight the significant biomarkers contributing to the latent variables of the autoencoder. For SCNV, biomarkers like 9p21.3 and 13q22.1 exhibit positive SHAP values (0.0170 and 0.0169), indicating their contributions towards increasing the value of the latent variable. Conversely, biomarkers such as 4q12 and 9q34.3 demonstrate negative SHAP values (−0.0243 and −0.0262), suggesting their role in decreasing the value of the latent variable. Furthermore, the magnitude of SHAP values for the top 10 features signifies the strength of the feature’s impact on the latent variable. In miRNA, hsa-mir-922, hsa-mir-3115, and hsa-mir-320c-2 display positive SHAP values (0.048, 0.025, and 0.017), signifying their positive influence on latent variables. However, hsa-mir-3129 stands out with a negative SHAP value (−0.020), indicating its suppressive effect on latent variables. In RNAseq analysis, genes like MEOX1 show positive SHAP values (0.001), suggesting their role in increasing the latent variables, albeit with relatively smaller impacts compared to other omics layers. Lastly, in methylation, interestingly, all top 10 biomarkers display positive SHAP values indicating their contribution to increasing the value of the latent variable. These SHAP values provide a nuanced understanding of biomarkers’ contributions, offering insights into the underlying biological mechanisms and potential targets for therapeutic intervention.

#### Biomarkers impact using SHAP for breast

**Figure 3:**
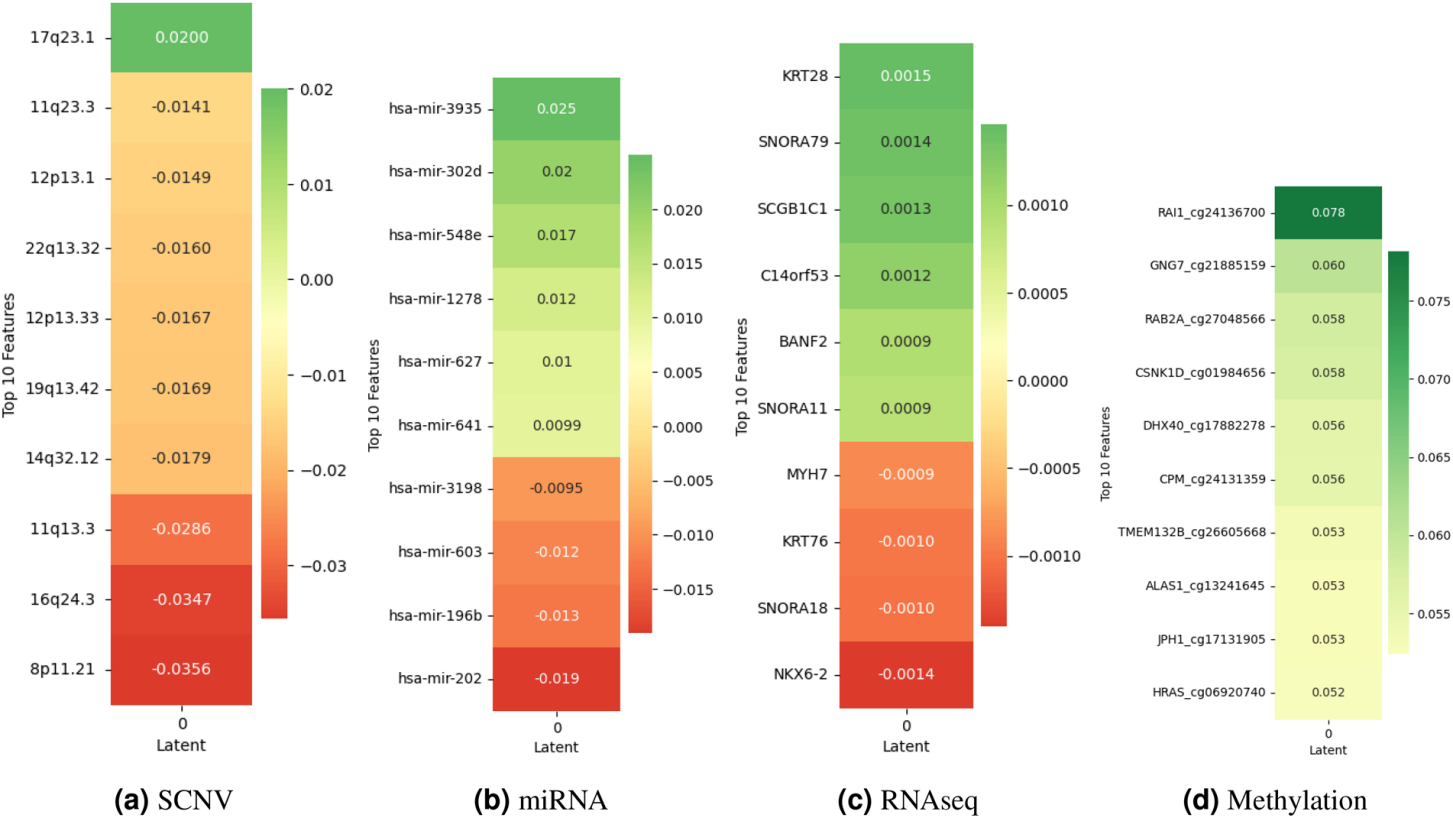
SHAP values demonstrate the impact of biomarkers on contributing to the latent variables of the autoencoder in Breast multi-omics data.

In Fig. 3, in the Breast cancer dataset, SCNV reveals distinct impacts on Breast cancer predictions. Notably, biomarker like 17q23.1 display positive SHAP values (0.020), suggesting their role in increasing the value of the latent variable. Conversely, biomarkers such as 11q23.3 and 12p13.1 exhibit negative SHAP values (−0.0141 and −0.0149), indicating their contribution to decreasing the value of the latent variable. In miRNA, biomarkers such as hsa-mir-3935 and hsa-mir-302d display positive SHAP values (0.025 and 0.020) and biomarkers such as hsa-mir-196b and hsa-mir-202 show negative SHAP values (−0.013 and −0.019). In RNA-seq, genes like KRT28, SNORA79, and SCGB1C1 demonstrate positive SHAP values, implying their positive influence on latent variables. Conversely, genes like MYH7 and KRT76 exhibit negative SHAP values, suggesting their suppressive effects. Lastly, in methylation, interestingly, all top 10 biomarkers display positive SHAP values, implying their contribution to increasing the value of the latent variable.

### Survival analysis for glioma

**Figure 4:**
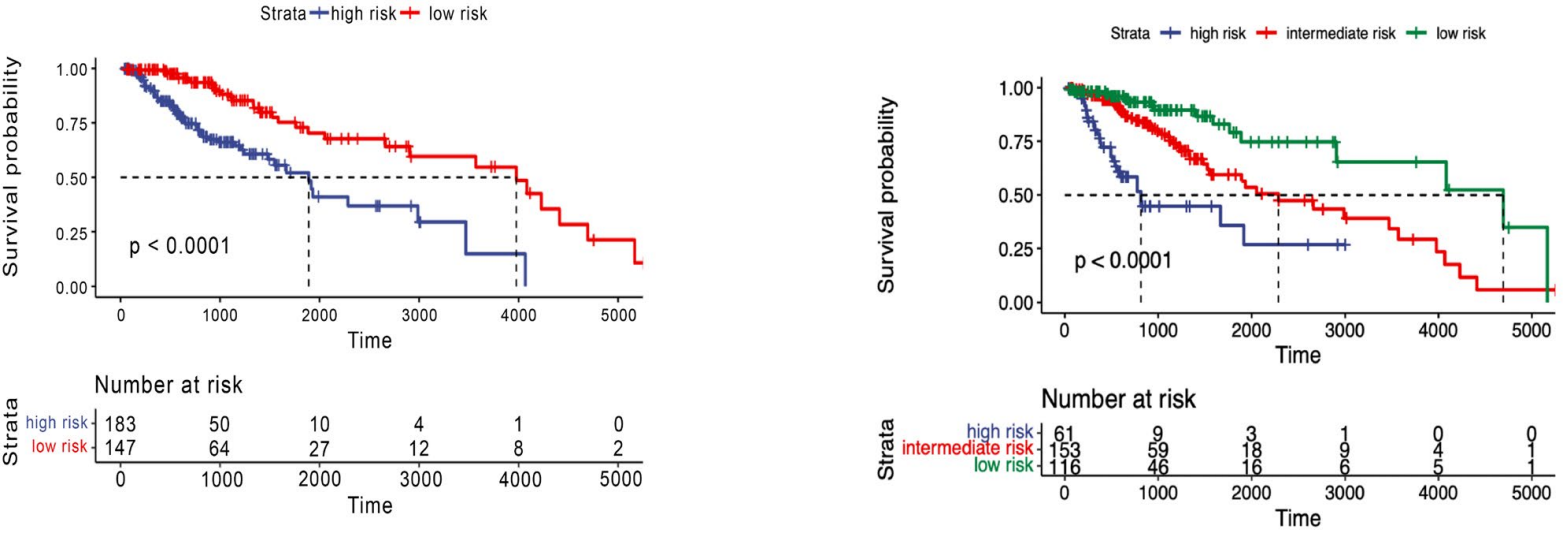
Overall survival of our method on Glioma patients in training set stratified by hierarchical clustering using multi-omics latent variables. The ‘*p*’ value represents the *p*-value of the log-rank test comparing the different groups.

**Figure 5:**
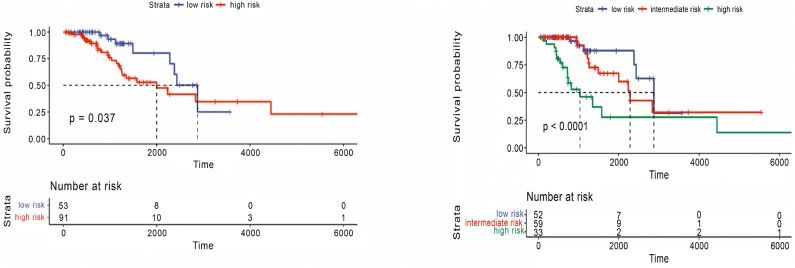
Overall survival of our method on Glioma patients in the testing set stratified by hierarchical clustering using multi-omics latent variables. The ‘*p*’ value represents the *p*-value of the log-rank test comparing the different groups.

We investigate whether the patients can be stratified into risk groups for Glioma cancer using the latent features from the multi-omics genomics data. The latent features are learned from our proposed framework, as shown in Fig. 1. First, for each type of cancer, the data is decomposed to 70% training data for model building and 30% testing data. Hierarchical clustering divides the patients into two or three risk groups. Then, to evaluate the ability of the multi-omics latent features to stratify patient overall survival (OS), a univariate regression model is fitted across Glioma patients in the training set (N=330) and testing set (N=144). The significance levels are indicated as $-log_{10}$ (*p*-value). Kaplan-Meier curves visualize the probability of survival outcomes over time in each group as shown in Figs. 4 and  5. A general observation is revealed from the results that multi-omics latent variables are significantly associated with patient OS in univariate models across all the patients in the training set. The patients could be stratified into low (N=147) and high-risk (N=183) groups and three groups with significantly different OS (*p*-value<0.05) as shown in Fig. 4.

**Figure 6:**
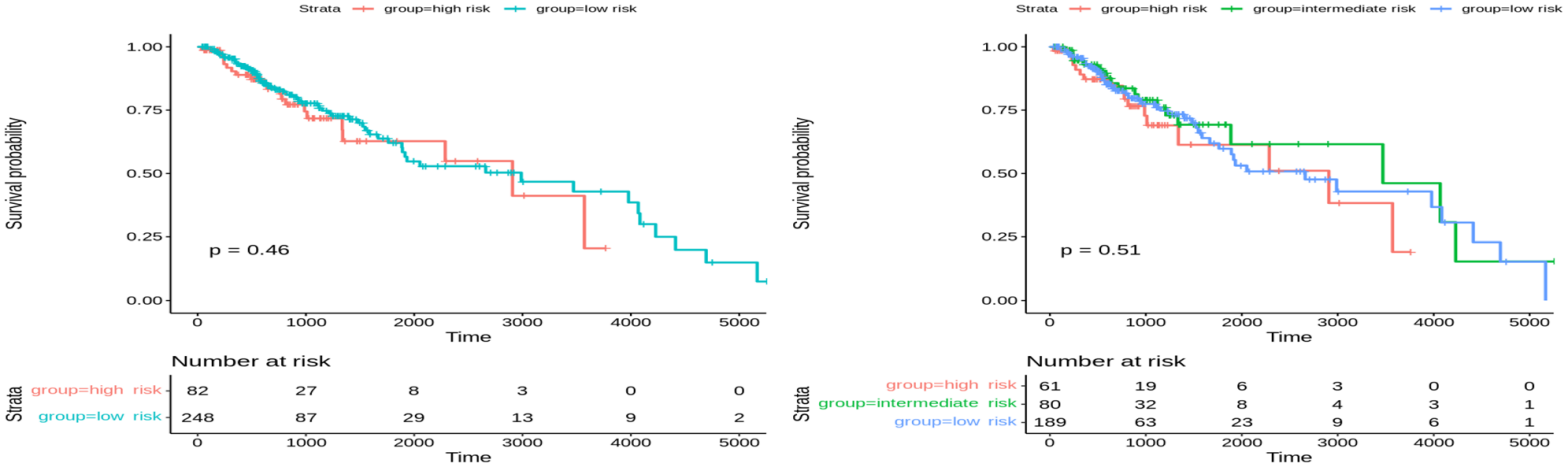
Overall survival of MOFA on Glioma patients in training set using multi-omics latent variables. The ‘*p*’ value represents the *p*-value of the log-rank test comparing the different groups.

**Figure 7:**
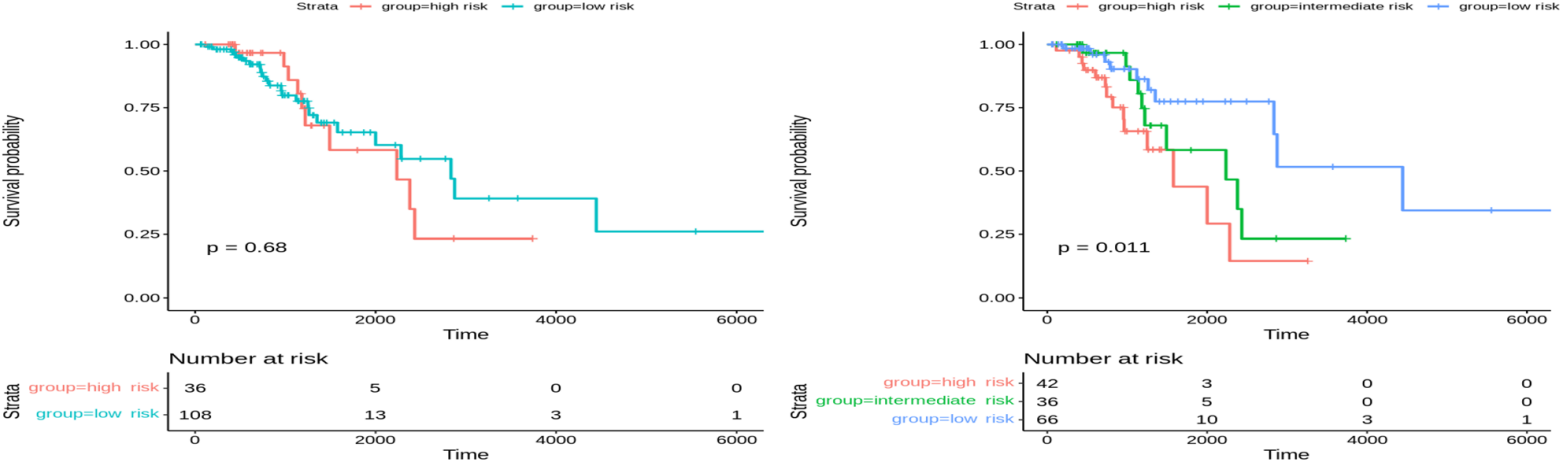
Overall survival of MOFA on Glioma patients in the testing set using multi-omics latent variables. The ‘*p*’ value represents the *p*-value of the log-rank test comparing the different groups.

In the test set of Glioma cancer, significant results were observed when clustering the data into two and three groups (Fig. 5). A *p*-value of 0.037 was obtained when clustering into two risk groups and less than 0.0001 for three risk groups. Compared with the state-of-the-art method, MOFA, the latent factors extracted from both training and testing sets did not significantly stratify patients into two or three risk groups (Figs. 6 and 7). The *p*-value was insignificant in all training and testing sets except for the Glioma testing set. These results were obtained using five factors that yielded the best performance using the MOFA method. Therefore, our framework can generate important latent features from multiple genomics data on patients’ overall survival. The clustering model can dichotomize patients with statistically significant *p*-values across all Glioma patients.

### Survival analysis for breast cancer

**Figure 8:**
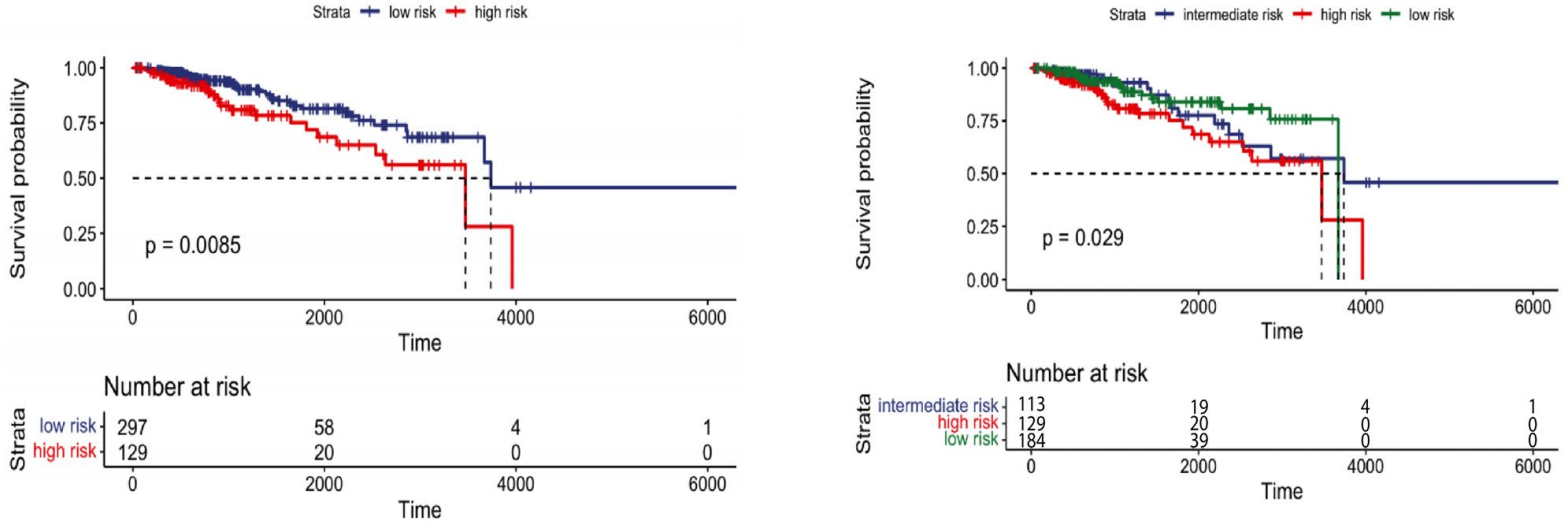
Overall survival of our method on Breast patients in training set stratified by hierarchical clustering using multi-omics latent variables. The ‘*p*’ value represents the *p*-value of the log-rank test comparing the different groups.

**Figure 9:**
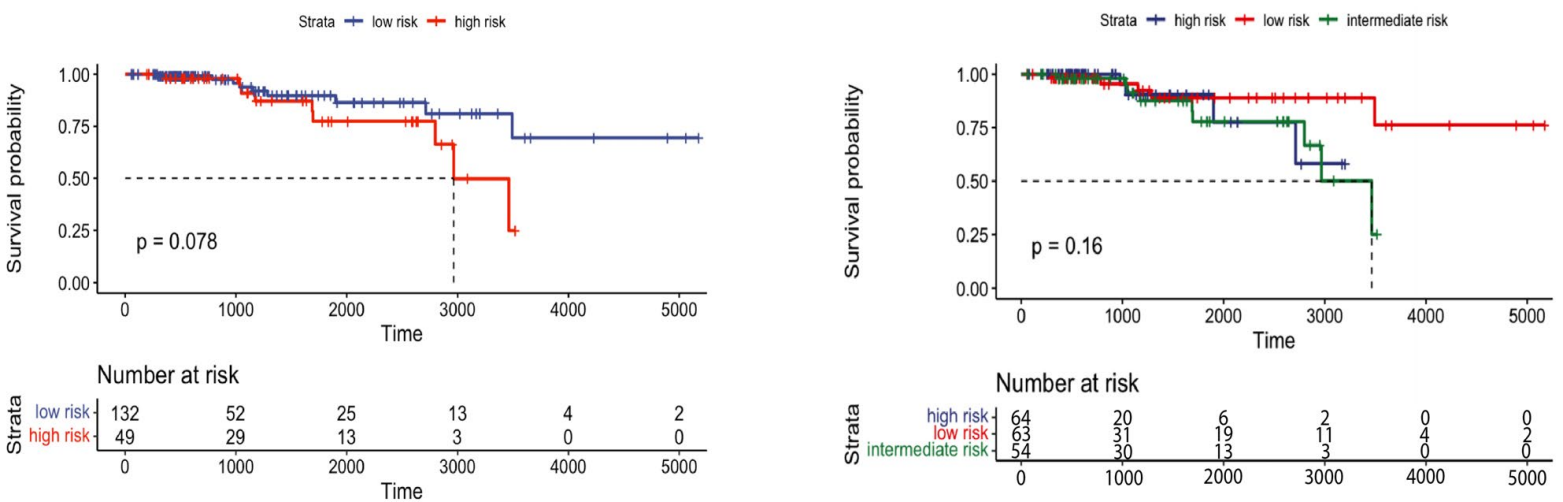
Overall survival of our method on Breast patients in the testing set stratified by hierarchical clustering using multi-omics latent variables. The ‘*p*’ value represents the *p*-value of the log-rank test comparing the different groups.

**Figure 10:**
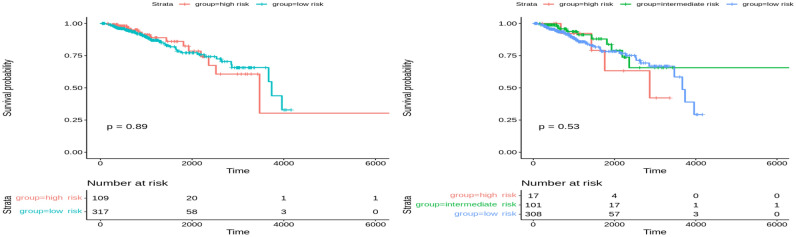
Overall survival of MOFA on Breast patients in training set stratified by hierarchical clustering using multi-omics latent variables. The ‘*p*’ value represents the *p*-value of the log-rank test comparing the different groups.

**Figure 11:**
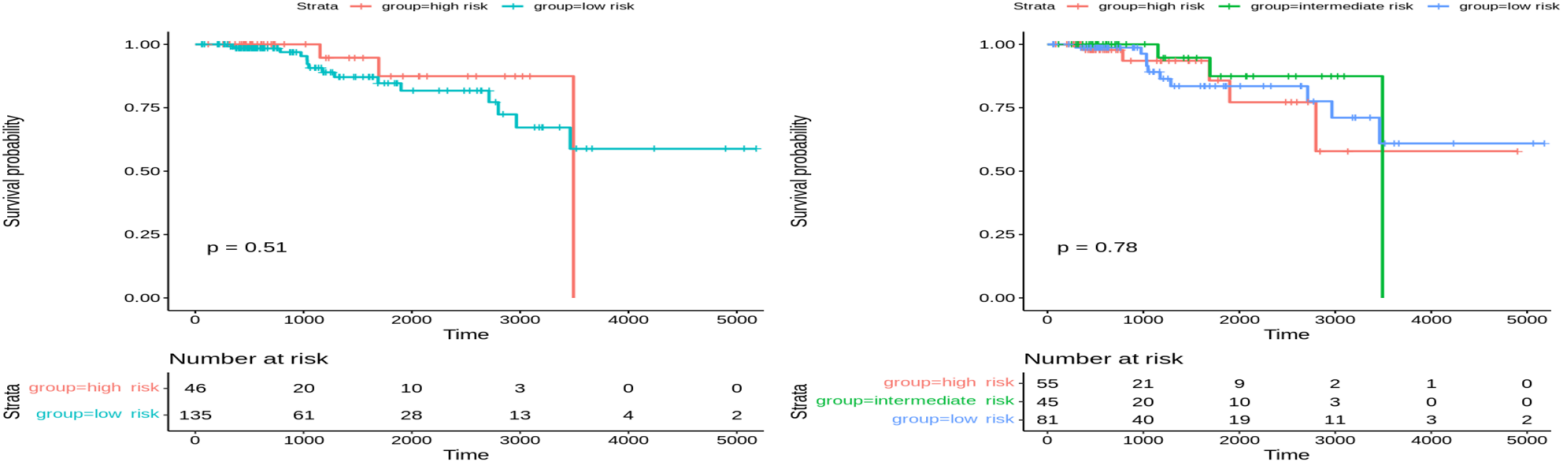
Overall survival of MOFA on Breast patients in the testing set stratified by hierarchical clustering using multi-omics latent variables. The ‘*p*’ value represents the *p*-value of the log-rank test comparing the different groups.

In the training set of Breast cancer (N=426), patients were stratified into two and three groups using learned latent variables from multi-omics data. Hierarchical clustering revealed a significant difference between the two risk groups (*p*-value=0.0085) and for three groups (*p*-value=0.029). Survival curves are shown in Fig.8. However, the testing set (N=181) patients couldn’t be stratified into risk groups with significant differences. Kaplan-Meier survival curves of the risk groups didn’t show significant differences at the 5% significance level between the two and three curves (*p*-value=0.078 and 0.16, respectively), as shown in Fig.9. Our method outperformed MOFA in significantly stratifying Breast cancer patients into multiple risk groups. This was observed by developing a clustering model that dichotomized Breast cancer patients using the latent factors of the MOFA method. As shown in Figs. 10 and 11, there was no statistically significant difference between the two and three risk groups across all Breast cancer patients in both the training and testing sets.

**Figure 12:**
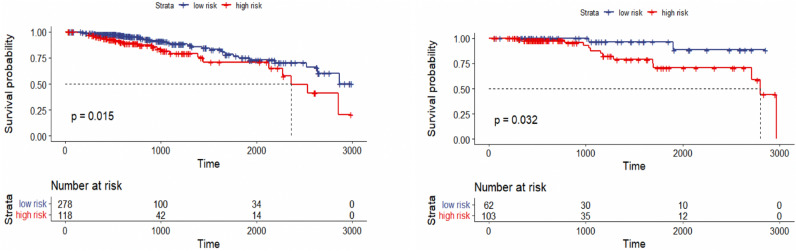
Overall survival of our method on Breast patients in the training and testing sets with restriction survival time to 3000 days, stratified by hierarchical clustering using multi-omics latent variables. The ‘*p*’ value represents the *p*-value of the log-rank test comparing the different groups.

Since very few patients can survive longer than 3000 days, to obtain more significant results, we limit patients’ survival time to 3000 days. Using the combination of maximum distance and ward linkage for hierarchical clustering, both training and test set results are significant. As shown in Fig. 12, a *p*-value of 0.015 is observed when clustering the Breast cancer training set into two groups, while a *p*-value of 0.032 is obtained for the test set.

Overall, the results for Glioma cancer outperform those for Breast cancer. They indicate that features extracted from autoencoder models remain significant after tensor decomposition, further validating the meaningful utilization of multi-omics data in determining patients’ risk for specific cancer types. Identifying patients’ risk levels can potentially increase overall survival rates by facilitating earlier interventions or selecting more effective therapies tailored to different tumor stages.

### Interpret latent variables using t-SNE visualization

Deep learning methods have shown remarkable success in our method of learning the non-linear representation of the data. However, one of the main challenges with deep learning methods is the interpretation of the learned features, including latent variables, which can be highly complex and abstract, making it difficult to interpret the meaning of individual latent variables. Latent variables represent underlying biological features that cannot be directly observed, but visualization techniques such as t-SNE can be used to interpret them graphically.

**Figure 13:**
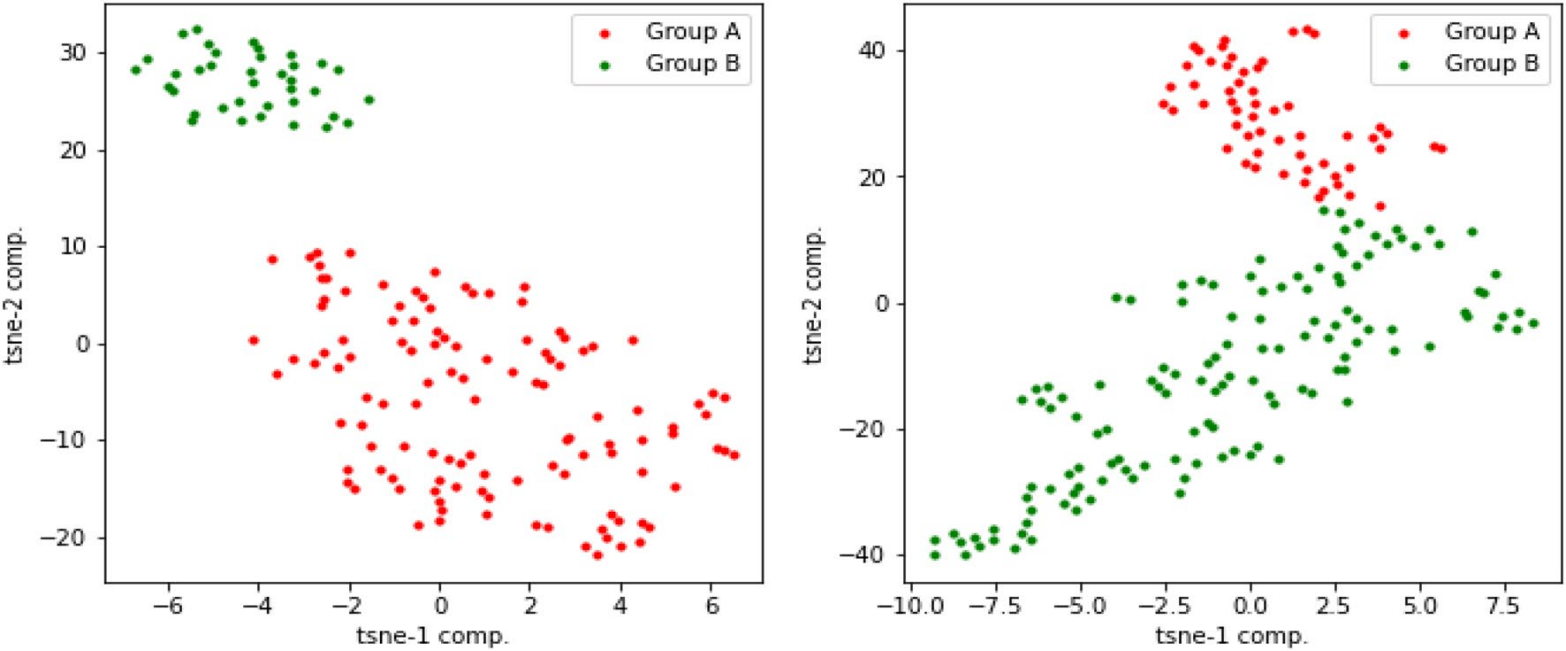
t-SNE plots of multi-omics latent variables in our method for Glioma and Breast testing data, respectively. Group A represents high-risk patients, and Group B represents low-risk patients.

**Figure 14:**
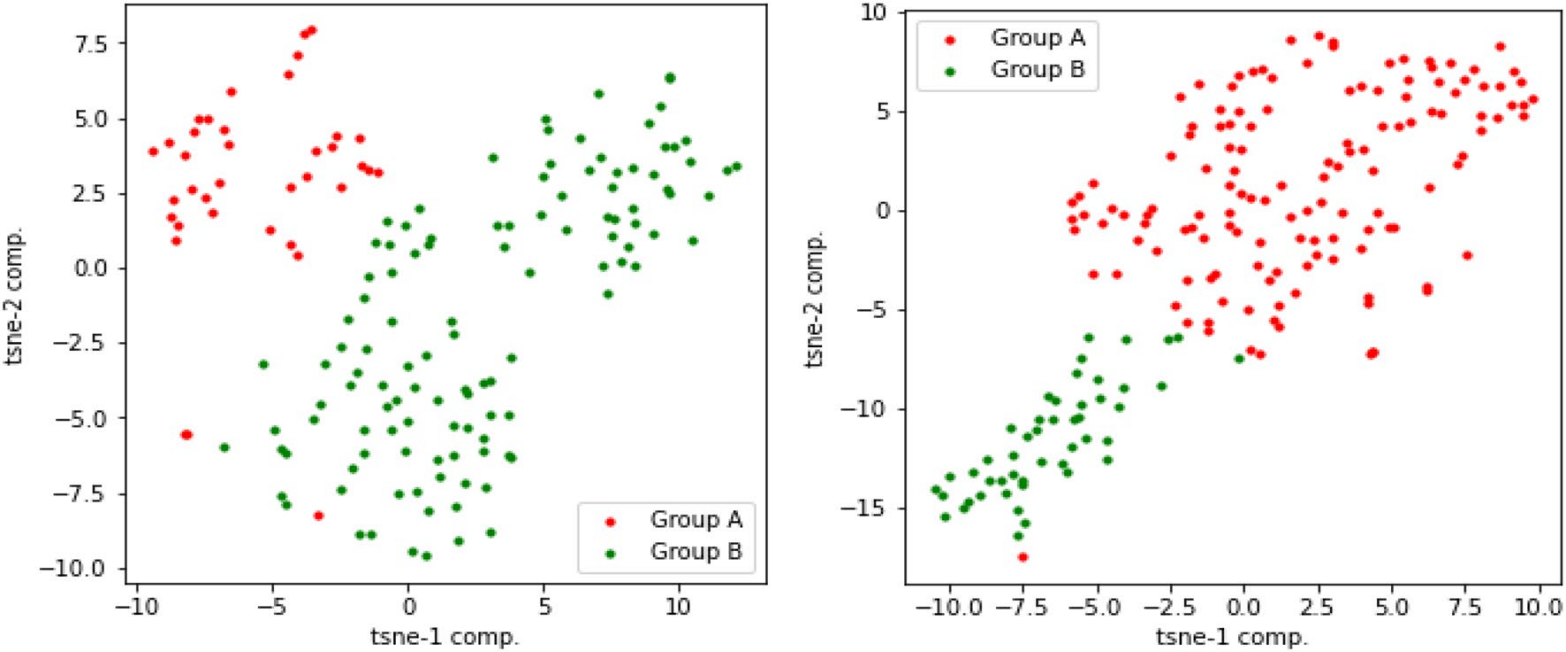
t-SNE plots of multi-omics latent variables in MOFA for Glioma and Breast testing data, respectively. Group A represents high-risk patients, and Group B represents low-risk patients.

In this experiment, we first applied t-SNE on the latent variables extracted from our proposed method to identify clusters of samples with similar latent variable values. As shown in Fig.  13, the resulting clusters can provide insights into the underlying biological processes or molecular pathways driving differences between samples. For example, they may suggest that the latent variables from multi-omics are capturing differences in gene expression or other molecular features associated with the disease subtype. Next, we evaluated the latent variables generated by MOFA for multi-omics. The results, visually observed in Fig.  14, demonstrate that our proposed method more effectively separates samples using multi-omics latent variables in the Glioma testing dataset.

**Table 1:** Classification of tumor purity on CP decomposition.

	Accuracy	Macro F1	Weighted F1
Logistic regression	0.59	0.37	0.44
KNN	0.41	0.29	0.23
Naive Bayes	0.59	0.37	0.44
Decision tree	0.41	0.29	0.23
SVM	0.59	0.37	0.44
Random forest	**0.69**	**0.60**	**0.65**
Gradient Boosting	0.44	0.36	0.32
Adaboost	0.59	0.37	0.44

The values in bold represent the highest accuracy.

**Figure 15:**
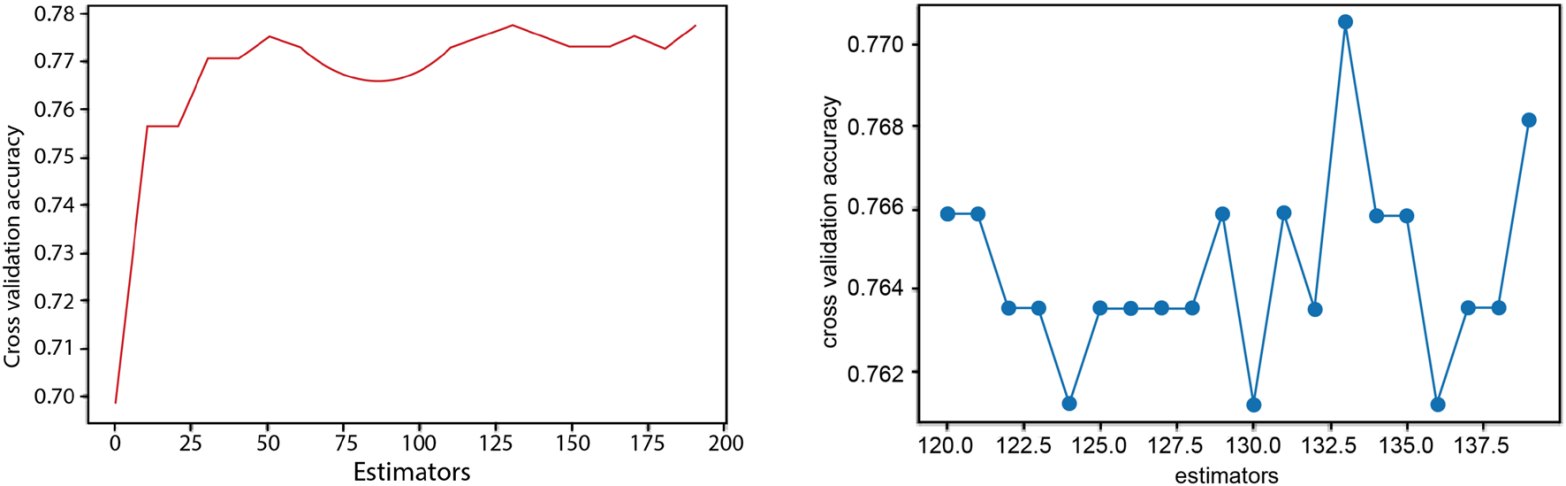
Parameter tuning.

### Revealing the biological insights of key biomarkers

We employed Gene Ontology (GO) Enrichment Analysis to investigate the impact of biomarkers on a latent variable for RNAseq omics. This reveals which biological processes, cellular components, or molecular functions are overrepresented^13–15^.

#### GO enrichment analysis for glioma

**Figure 16:**
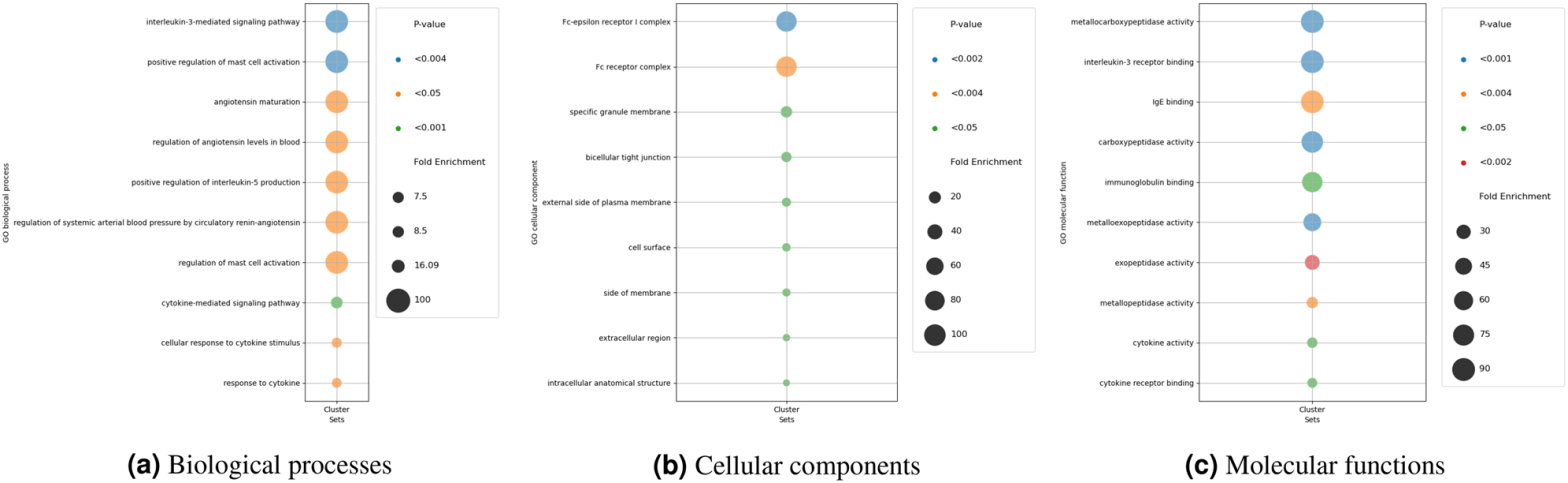
GO enrichment analysis results in Glioma dataset.

The analysis indicates significant associations between analyzed genes and various pathways for biological processes (Fig. 16), including interleukin-3-mediated signaling, positive regulation of mast cell activation, and angiotensin maturation. High Fold Enrichment values suggest crucial roles of these genes in immune response and physiological processes, highlighting their potential importance in biological functions. Furthermore, the cellular component analysis highlights significant enrichments in cell surface, external side of plasma membrane, and specific granule membrane, indicating their crucial roles. Intracellular anatomical structure show lower enrichments, suggesting diverse cellular functions. Fc receptor complexe exhibit remarkable enrichment, emphasizing their importance in cellular interactions. Lastly, the molecular function analysis reveals significant enrichments across diverse activities. Metallocarboxypeptidase, carboxypeptidase, and metalloexopeptidase activities exhibit high enrichments, underscoring their roles in peptide processing. Interleukin-3 receptor and IgE bindings demonstrate remarkable enrichments, suggesting their pivotal involvement in immune responses. These findings shed light on key molecular processes within the biological system.

#### GO enrichment analysis for breast

**Figure 17:**
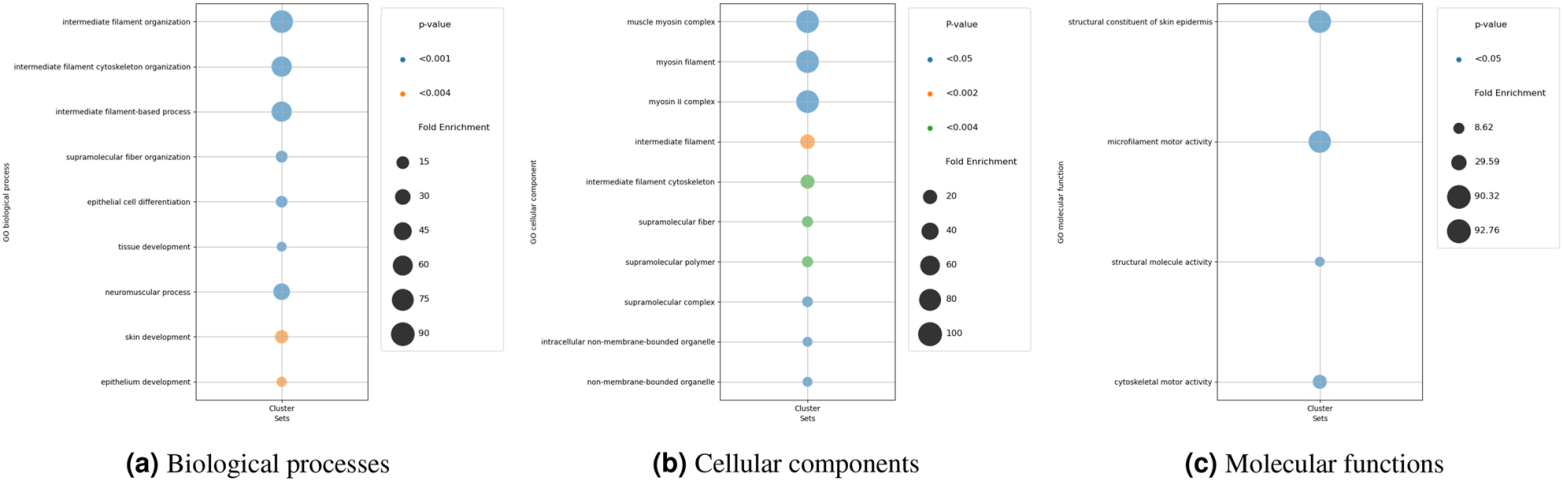
GO enrichment analysis results in Breast cancer dataset.

As shown in Fig. 17, the GO analysis unveils significant biological processes in the Breast cancer dataset, such as enrichments in intermediate filament organization and intermediate filament cytoskeleton organization, which underscore their pivotal roles in maintaining cellular structure. Supramolecular fiber organization and tissue development signify fundamental mechanisms driving tissue formation. Neuromuscular processes reveal critical interactions between nerves and muscles, essential for coordinated movement. The cellular component analysis highlights key cellular components: Intermediate filaments and cytoskeletons show high enrichments for structural support. Supramolecular fibers and polymers aid in cellular organization. Lastly, the molecular function analysis unveils notable enrichments in molecular functions: Structural constituents of skin epidermis and microfilament motor activities demonstrate high enrichments, crucial for skin integrity and cellular movement. Cytoskeletal motor activity plays a vital role in cellular transport and organization.

### Classification on tumor purity for CP decomposition of breast

We further evaluate the multi-omics latent variables to classify the patients based on tumor purity as presented in Table 1. We have conducted the experiments on the Breast data only because this feature exists only in its clinical data. Tumor purity is categorised into low and high groups based on a threshold of 0.7. Comparing the performance of different classification models based on the result sets of CP decomposition, it is observed that the model trained with the data after CP decomposition performs better. This classification problem is particularly challenging in the Breast dataset, and even state-of-the-art methods struggle to achieve high accuracy ^16^. Specifically, by testing the result sets of CP decomposition with different ranks, the best model is Random Forest which is trained on the result sets of CP decomposed by setting the decomposed rank to 9. The accuracy rate is 0.69, and the weighted F1-score is 0.65. For the remaining models, Logistic Regression, Naive Bayes, SVM, and AdaBoost have the same accuracy of 0.59, but their F1-score does not reach 0.4, so their performance is relatively poorer. Therefore, Random Forest can be used to classify tumor purity using the CP decomposition method.

Three parameters are tuned in the random forest model: estimators, max depth, and max features. Among them, estimators have the most significant impact on the results. The optimal values are identified using the search space. Firstly, we search estimator values from 0 to 200. A random forest is built based on the interval of 10, and the intervals are taken as the x-axis, and the corresponding cross-validation scores are set as the y-axis (Fig.15). The results show the highest accuracy value when the estimator is 133. Finally, using the estimators of 133 as the determining parameter, optimal values of both max depth and max features can be obtained in a similar process, which is two and nine, respectively.

## Discussion

We first emphasize the significance of utilizing multi-omics data and integrating latent variables using tensors to identify cancer risk groups. In this section, we perform experiments using single-omics data on Breast cancer. Patients are clustered into two groups, and survival analysis is conducted separately to assess the significance of features extracted from autoencoder models of single-omics. The *p*-values are reported in Table 2, indicating insignificant differences between risk groups when using single-omics separately across all technologies for Breast cancer data. Conversely, survival analysis results on Glioma single-omics data are significant, as shown in Table 3. Thus, the performance of latent variables from single-omics datasets of Glioma aligns with those of multi-omics, demonstrating the significance of using features from multiple omics in both Breast and Glioma datasets.

**Table 2:** *p*-value to stratify the patients into two risk groups for training and testing Breast data on single-omics.

	Training	Testing
RNAseq	0.25	0.28
SCNV	0.58	0.43
miRNA	0.15	0.58
Methylation	0.48	0.094

**Table 3:** *p*-value to stratify the patients into two risk groups for training and testing Glioma data on single-omics.

	Training	Testing
RNAseq	<0.001	0.013
SCNV	<0.001	0.004
miRNA	<0.001	0.04
Methylation	<0.001	0.005

Overall, our proposed framework offers several advantages. Firstly, it handles each single-omics dataset separately instead of combining them all at the outset. While concatenation-based integration methods have shown success in some cases, they often lead to the loss of information from smaller omics datasets. For instance, in our case, SCNV data, with a feature size of only 69–72, risks losing information when combined with larger datasets like methylation, which boasts over 330k features. Our approach mitigates this risk by processing each omics dataset individually before fusion. By implementing autoencoders for each omics dataset, we can effectively compress the original information. Secondly, rather than imposing a common target dimension for all omics datasets, we compress each dataset to its optimal size. This approach aims to retain the maximum amount of information from each single-omics dataset during the compression process. Furthermore, integrating multi-omics data into a large matrix can further exacerbate the loss of information from smaller-sized omics datasets during feature extraction or selection methods. To address this, we compress each omics dataset separately using autoencoders before integration, thereby reducing the risk of information loss. Our framework maximizes information retention for each omics dataset and then integrates them into a tensor, minimizing overall information loss and avoiding issues associated with handling multi-omics integration when datasets have significant size differences.

Objectively, the framework does not add any knowledge from the biological area. We aim to investigate the biological interpretation of the difference between the low and high-risk groups identified by the latent variables extracted from multi-omics cancer data, which could include

- Differential activation of oncogenic pathways: The latent variables may capture differences in the activation of pathways involved in cancer development and progression. Patients in the high-risk group may have higher levels of activation of oncogenic pathways, leading to more aggressive tumor growth and a worse prognosis.
- Immune system dysfunction: The latent variables may be associated with differences in the immune response to cancer. Patients in the high-risk group may have immune system dysfunction, such as reduced immune surveillance or an immunosuppressive tumor microenvironment, which allows the tumor to evade detection and destruction by the immune system.
- Treatment response: The latent variables may predict how well patients respond to different cancer treatments. Patients in the high-risk group may be less responsive to standard treatments, leading to a worse prognosis.

## Methods

### Data collection

The data used in this study were collected from an open platform LinkedOmics ^17^, which provides access to multi-omics data from all 32 TCGA Cancer Types and 10 Clinical Proteomics Tumor Analysis Consortium (CPTAC) cancer cohorts. The original data used in this study, the Glioma (https://gdac.broadinstitute.org/runs/stddata__2016_01_28/data/GBMLGG/20160128/)  and Breast (https://gdac.broadinstitute.org/runs/stddata__2016_01_28/data/BRCA/20160128/)  Invasive Carcinoma datasets, are freely available on the Firehose platform of the Broad Institute (http://gdac.broadinstitute.org/). To ensure enough samples to support our training and testing, we selected the Glioma and Breast Invasive Carcinoma cancer types as they have more than 1000 samples available. Four omics data have enough samples and significant differences in the size of the features for each cancer type. The four omics selected for Breast are methylation (CpG-site level, HM450K), miRNA (HiSeq, Gene level), RNAseq (HiSeq, Gene level), and SCNV (Focal level, log-ratio). Similarly, in Glioma, the same omics are chosen except for changing the miRNA to miRNA (Gene level) as there is no miRNA (HiSeq, Gene Level) in the available data. All the omics contain continuous data only as researching on mixture data type is out of scope for this study. The selected omics data include portions of the shared samples across the four technologies and clinical information. To ensure that the same set of common samples was used in the experiments, we matched each of the four technologies’ data with the corresponding clinical data to define our dataset. Consequently, we obtained 616 common raw samples for Breast cancer and 508 for Glioma.

The dimensions of the omics data varied significantly, with the largest being methylation, which had up to 335,854 dimensions, and the smallest being SCNV, with only 69 dimensions. RNAseq had 20,155 dimensions, and miRNA had 823 dimensions. The huge dimension difference makes handling data loss and delusion during dimension matching challenging. All the values in omics are continuous data. Further, we noticed many features only contained values for a few samples while all others were zero. The collected omics data of both cancer types have two common challenges. The first challenge is the huge size difference between different omics. For example, SCNV has only 69 genes, while methylation can have more than 330 thousand genes. This difference does not allow combining the data because the larger ones may dilute the lower-size omics. The other challenge is the low number of samples after the common samples are selected across various omics and the huge number of dimensions in some technologies, such as methylation.

### Our framework

Our proposed framework consists of three main components, as shown in Figure 1. Firstly, the original omics data is dimensionalised using autoencoders, which employ a combination of non-linear functions to reconstruct the original input. It is known that this method performs well when applied to biological data, with less information lost^5,7,18–20^ and is therefore well suited to handle omics information. Secondly, the processed multi-omics data is fused into a tensor. The significant global features of different omics datasets can be learned through CANDECOMP/PARAFAC (CP) decomposition to extract interpretable latent factors. While the original data may not be fully recoverable from the CP decomposition of the compressed data, we focused on obtaining a more interpretable and meaningful representation of the data that captures its essential characteristics. Finally, the components extracted from tensor decomposition are utilized for clustering. The clustering results are evaluated using survival analysis. Additionally, a supervised learning model is built and used to predict Tumor Purity for the Breast dataset due to the availability of class labels.

#### Data preprocessing

The collected datasets are split into 70% training data and 30% testing data to ensure a sufficient number of test cases. Data cleaning is applied to Breast and Glioma datasets to handle the missing values. Only methylation contains a limited number of missing values, so we replace them with the mean value of the related gene features^21,22^. Since it is uncertain whether zero values in omics data are meaningful, we decided to keep them to avoid any loss of meaningful information^23^. After the cleaning phase, the data is scaled by the MinMaxScaler function as follows

$$x^{'}=\frac{x-min(x)}{max(x)-min(x)}$$

#### Feature extraction using stacked autoencoder

Since the sizes of omics data are various and can be extremely large due to many genes^21, 24, 25^, it is necessary to reduce or compress them to a reasonable size. We aim to keep the maximum information in the extracted features from all the omics datasets. To achieve this goal, the stacked autoencoder model is implemented and applied to separate omics. It consists of an artificial neural network widely used for dimension reduction. It aims to extract meaningful information from the input dataset, transform them into smaller size latent and reconstruct the input data from the latent^26^. To create the stacked autoencoder model, we have implemented the following steps:

**Step 1: Encoding**. Given an omics dataset, *D* with *N* samples and *d* features, an encoder in the autoencoder model compresses the *d* features into $d'$ where d >d’. The hidden layers stack within the encoder, reducing nodes between *d* and $d'$. The encoder part uses a non-linear mapping function to map the input data to hidden layer units and between the hidden layers. Assume *h* denotes the activation of the hidden layer neural unit, then its mathematical expression is as follows

1 $$\begin{aligned} h=f(x) = S_{f}(wx+p) \end{aligned}$$

where *w* represents the learning weighted matrix connecting the input layer and the stacking hidden layers. $S_{f}$ is the activation function at the last hidden layers, which is usually a Sigmoid function or a Tanh function as shown below in Eq. (2) and Eq. (3) respectively.

2 $$\begin{aligned} f(x) = \frac{1}{1+e^{-x}} \end{aligned}$$

3 $$\begin{aligned} f(x) = \frac{e^{x}-e^{-x}}{e^{x}+e^{-x}} \end{aligned}$$

We also add a ReLU activation function in each hidden layer which has the following equation

4 $$\begin{aligned} ReLU(x) = (x)^{+} = max(0,x) \end{aligned}$$

The following section shows the structure of the encoder for both datasets: Breast Invasive Carcinoma and Glioma.

**Figure 18:**
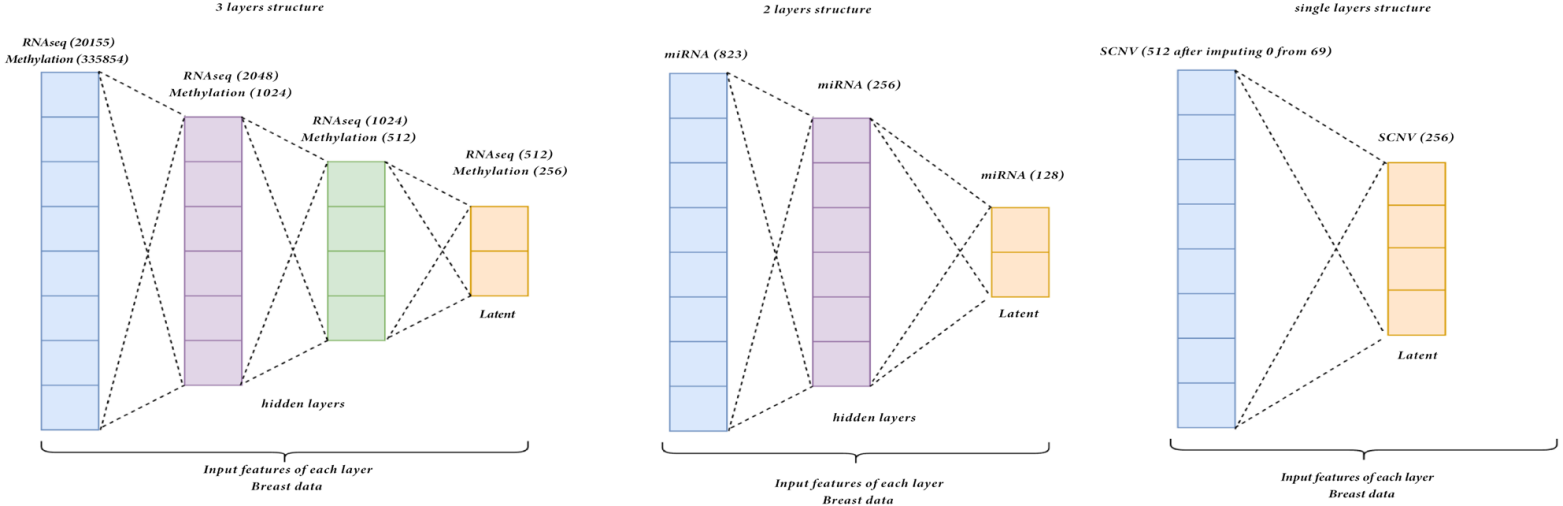
Encoder Structure for Breast Invasive Carcinoma.

**Encoder Structure for Breast Invasive Carcinoma**. After preprocessing, the four selected omics datasets for Breast cancer data contain the same sets of samples but vary in features. These include SCNV, miRNA, RNAseq, and methylation, comprising 69, 823, 20,155, and 335,854 features, respectively. Each omics dataset is processed by a separate autoencoder, except for SCNV. Given its small size relative to the others, SCNV is imputed with zero values to match the dimensions of the other datasets, ensuring no significant reduction in their sizes. SCNV is imputed to 512 features and incorporated into the autoencoder. To prevent dilution of information due to zero values, specific values for each layer are selected within a defined range, halving the dimension of each successive hidden layer. Hardware limitations restrict the first hidden layer to a maximum of 1024 nodes for the two larger omics datasets. Consequently, determining an optimal target latent feature size below 1024 becomes necessary. Each hidden layer is connected by a ReLU activation function. The two larger omics datasets are processed by a three-layer encoder with specific hidden nodes, while miRNA is processed by a two-layer encoder, and SCNV by a single-layer encoder, as depicted in Fig. 18

.

**Figure 19:**
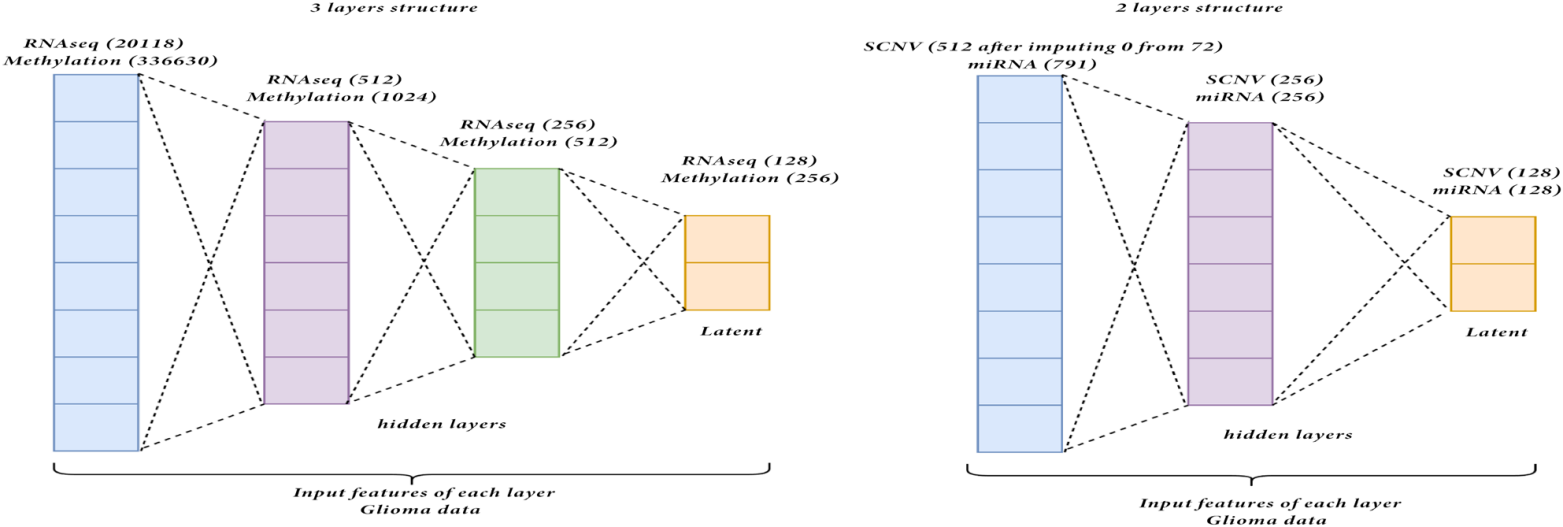
Encoder structure for Glioma.

**Encoder Structure for Glioma**: The same four omics in the Breast data are selected for Glioma, with minor differences in the feature size. Initially, SCNV has 72 features, miRNA has 791, methylation has 336630, and RNAseq has 20118 features. Since these omics’ feature sizes are similar to those in Breast data, encoders with similar structures are implemented. During the evaluation through training loss, there are changes in the target latent size and the features in the hidden layers for some omics. Similar to the methylation in the Breast, it cannot increase the output features to more than 1024 of the first hidden layers due to hardware limitations. Hence, it is compulsory to select the optimal target latent feature size below 1024. More specifically, there is a ReLU activation function between each hidden layer. Similar to the ones in Breast data, the two large omics are served by the three-layer encoder, while the other two smaller omics fit into the two-layer encoder. The detailed structure is presented in Figure 19.

**Step 2: Bottleneck**. The compressed output is generated in the latent space in the bottleneck layer, having the same feature size as the number of nodes in the last hidden layer of the encoder. This latent output is regarded as the compressed output of the model. There are two usages of this latent output. The first usage is to put into the decoder of the stacked autoencoder to reconstruct the original input and evaluate the model by calculating the loss between the original input and reconstructed output. The other usage is to take this latent as the model output for the next component of our framework. For Breast Invasive Carcinoma, the optimal latent size for each is selected by inspecting the training loss and the validation loss in 10-fold cross-validation and gaining the one with the lowest training loss and stable low validation loss. After evaluating different targets, e.g. latent feature sizes, including 64, 128, 256, 512 and training the model using the entire train set, the resulting latent for each omics are 256 for SCNV, 128 for miRNA, 256 for methylation and 512 for RNAseq. For Glioma, similar to the Breast cancer data, the evaluation of the optimal latent feature size is performed similarly using 10-fold cross-validation. As a result, the optimal latent for each omics is 128 for SCNV, 128 for miRNA, 256 for methylation and 128 for RNAseq.

**Step 3: Decoding**. The decoder part of the model mirrors the encoder part. Setting the same numbers of hidden layers, the decoder aims to reconstruct the input from the latent as follows

5 $$\begin{aligned} y = g(h) = S_{g}(wh+q) \end{aligned}$$

where the *w* represents the weight matrix between hidden layers, *q* is the bias term, *y* represents the reconstructed input and $S_{g}$ represents the activation function for the decoder.

**Step 4: Loss function and back-propagation**. To calculate the loss between the original input and reconstructed output, Mean Squared Error(MSE) is the loss function commonly used for autoencoder training. Assuming input *x* and target *y*, the loss can be written as

6 $$\begin{aligned} l(x,y) = L = {l_{1},...,l_{N}}^{T}, l_{n} = (x_{n}-y_{n})^{2} \end{aligned}$$

where *N* is the batch size 128. Since the default setup of the model is used

$$\begin{aligned} l(x,y)= mean(L) \end{aligned}$$

**Parameter setting**. The autoencoder is trained using 10-fold cross-validation to determine the optimal target latent size. Using the entire train set, each model will be trained again by setting the latent output as the optimal value. After running ten epochs, both the average training loss and validation loss of each model are around 1% to 3%. Adam is selected as the optimizer, and the learning rate is set to 0.001 to avoid overlearning. The trained models generate the latent for each test set.

#### Multi-omics tensor data fusion and decomposition

**Tensor Data Fusion. ** A 3D tensor is used to fuse these latents of each omics data. However, The matrices in the tensor must have the same size. To retain most of the information fused into the tensor, the latent embeddings with larger sizes are divided into smaller embeddings with the same size as the smallest latent embedding. These matrices are the same size, so they can be stacked to form a tensor. The stacking strategy frequently merges multiple data sources into a single tensor that can be utilized in machine learning models^27,28^. The quality of the extracted features impacts the effectiveness of this approach. If the features are noisy or not relevant to the intended task, then the stacking strategy may not be effective. However, in our method, we implement an autoencoder to compress the data and learn new features using non-linear functions.

**Figure 20:**
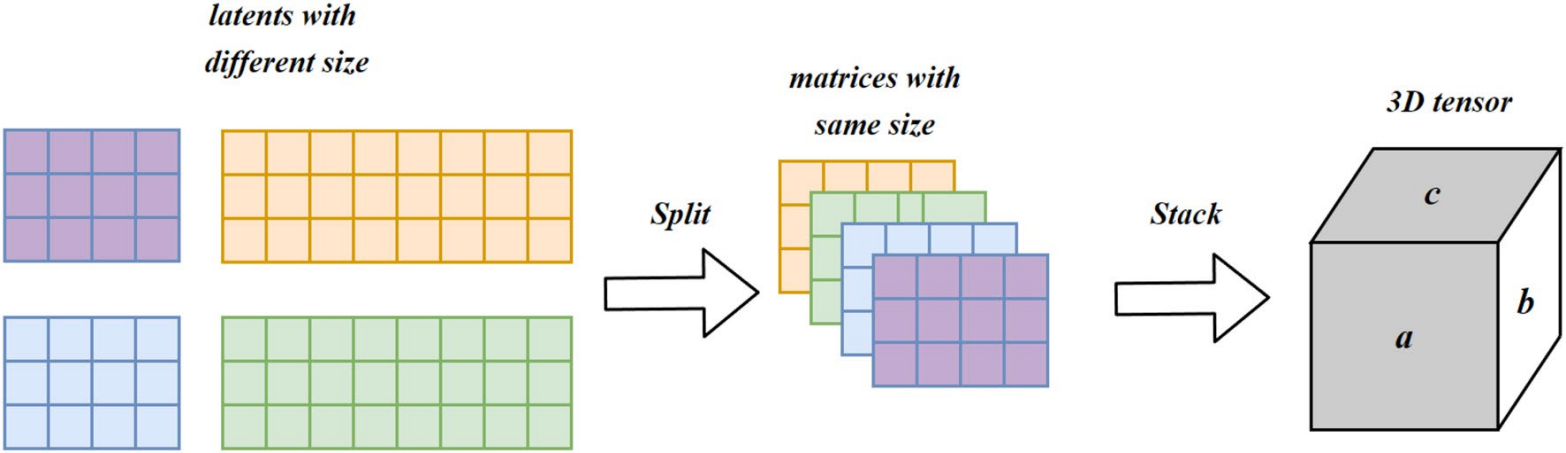
Stacking the matrices with the same size to build the tensor of shape: samples, assays, and latent features.

Four sets of latent features for Breast training data are created, which contain the following shapes (samples, latent features) among various omics: SCNV (431,256), miRNA (431, 128), RNAseq (431, 512) and methylation (431, 256). The test data contains the same feature size with 185 samples. To integrate these latents into the same size, the minimum size among these latents is set as the target and split the larger ones evenly to the target. For example, we split four pieces of RNAseq, each with a shape (431,128). Then, the pieces can be stacked to form a tensor, as demonstrated in Fig. 20. Therefore, it can fuse all the related data compressed by autoencoders into a tensor. After stacking them in the orthogonal axis, we successfully retrieve two tensors with shapes (431, 9, 128) and (185, 9, 128) for the train and test sets, respectively. Like the Breast data, four latents belonging to Glioma are generated after compressing the original by autoencoder. The shapes of each training set are as follows: SCNV (355,128), miRNA (355,128), RNAseq (355,128) and methylation (355, 256). The test sets share the same feature size separately and has 153 samples. After splitting to match 128, the minimum feature size, they are stacked in the orthogonal axis to form two tensors with shapes of samples, assays, and latent features, e.g., (355, 5, 128) and (153, 5, 128) for the train set and test sets respectively.

**Figure 21:**
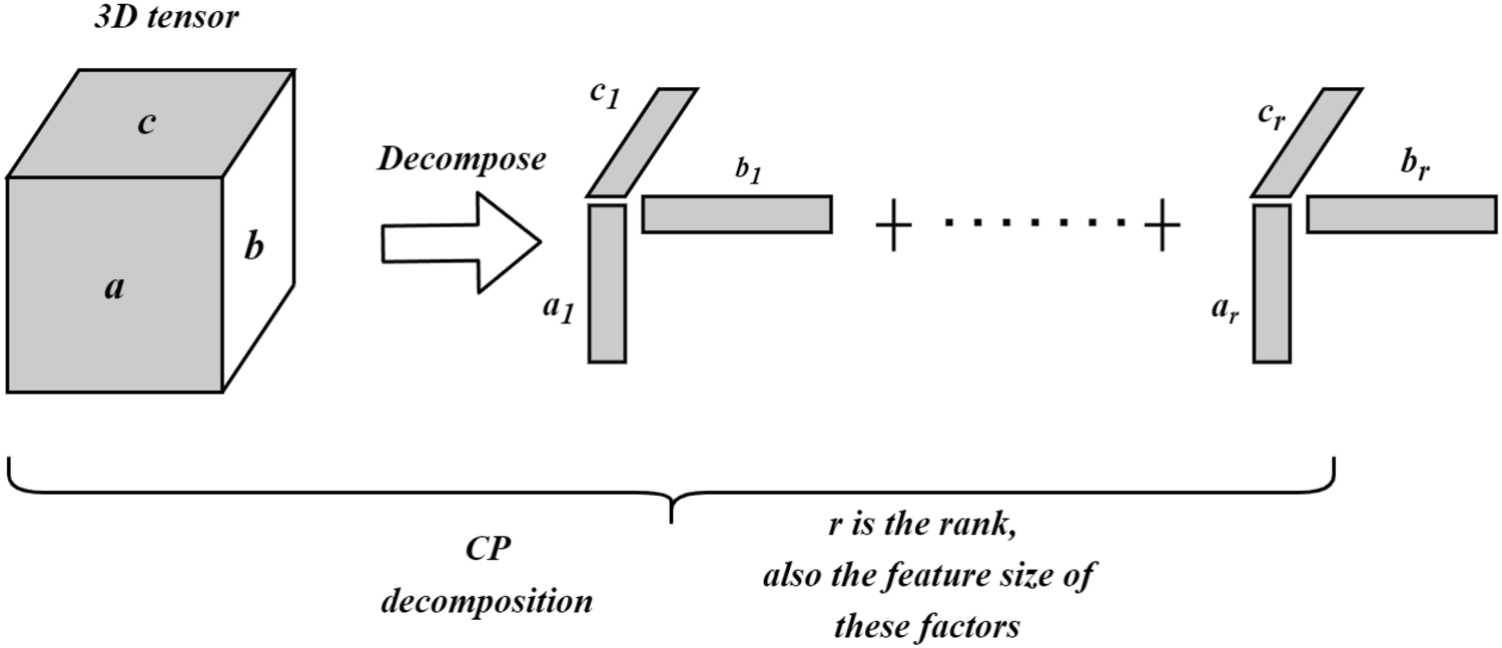
Tensor decomposition.

**Tensor Decomposition process. ** Given a tensor $X \in \Re ^{I \times J \times K}$, We use Parafac^29^ (a.k.a CP decomposition) to decompose the tensor into three matrices A, B and C as shown in Fig. 21. Matrix A represents the patient’s mode, B represents the omics feature mode and C represents the genes (latent features) mode. In this sense, a tensor *X* can be written as

7 $$\begin{aligned} X \approx \sum _{r=1}^R \lambda _r \ A_{r} \circ B_{r} \circ C_{r} \equiv [ \lambda ; A,B,C] \end{aligned}$$

where “$\circ$” is a vector outer product. *R* is the latent element, $A_{r}, B_{r}$and $C_{r}$ are r-th columns of component matrices $A \in \Re ^{I \times R}$, $B \in \Re ^{J \times R}$and $C \in \Re ^{K \times R}$, and $\lambda$ is the weight used to normalize the columns of *A*, *B*, and *C*.

The main goal of CP decomposition is to decrease the sum square error between the model and a given tensor *X*:

8 $$\begin{aligned} \min _{A,B,C} \Vert X - \sum _{r=1}^R \lambda _r \ A_{r} \circ B_{r} \circ C_{r} \Vert ^2_f, \end{aligned}$$

where $\Vert X\Vert ^2_f$ is the sum squares of *X*, and the subscript *f* is the Frobenius norm. In this work, we use the core consistency diagnostic technique (CORCONDIA) technique described in^11^ to determine the number of rank-one tensors *R* when it decomposed using the CP method.

The function presented in Equation (8) is a non-convex problem, as it seeks to optimize the sum of squares of three matrices. However, the problem can be reduced to a linear least squares problem by fixing two of the factor matrices and solving only the third one. Following this approach, the ALS technique can be employed, which iteratively solves each component matrix while keeping all other components fixed until convergence.

We note that ALS can yield sensitive solutions and is not generally robust, highlighting the need to incorporate penalty and regularization techniques. Introducing regularization and penalization parameters into the $L_1$ norms enables smoother representations of the outcome, mitigating issues related to local minima perturbations^30^. The algorithm for CP decomposition using regularized ALS (RALS) is outlined in Algorithm 1. The $L_1$ penalty terms $||X||{L_1}=\sum {\cdot }|x_{\cdot }|$ enforce sparsity in *X*.

**Algorithm 1:**
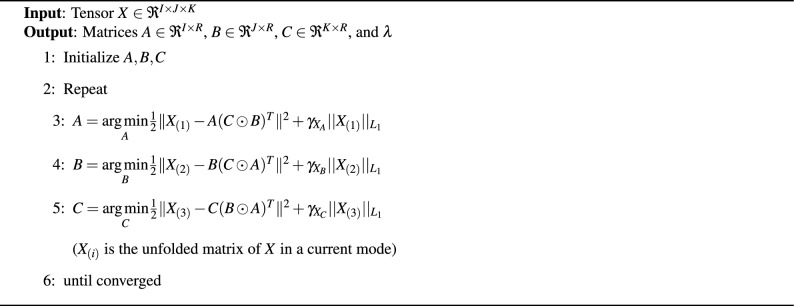
Regularized Least Squares for CP

**Figure 22:**
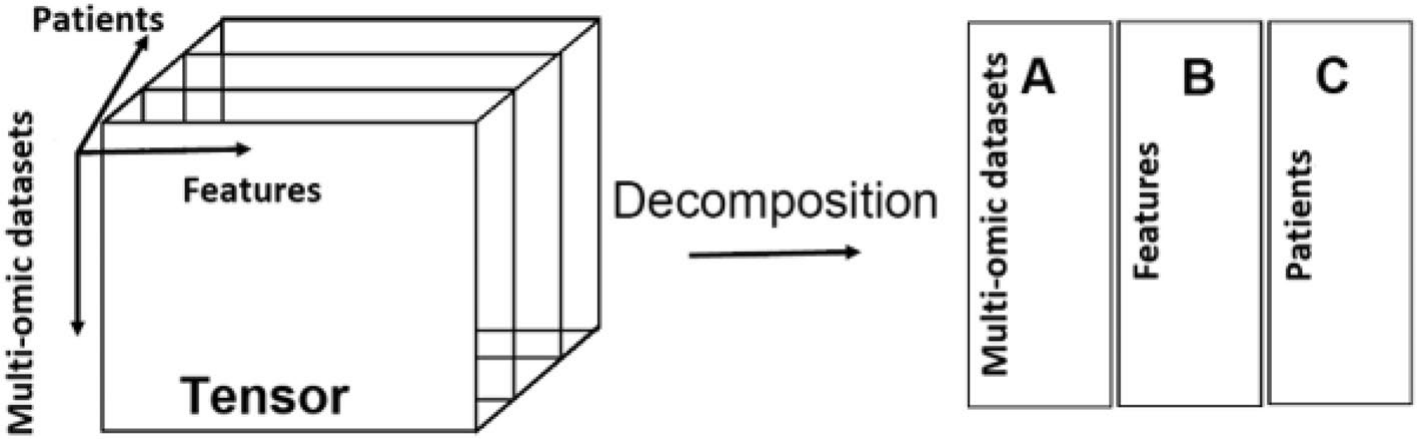
Multi-omics data fused in a tensor.

Interestingly, our proposed method employs a tensor for data fusion. The alternative naive approach would simply concatenate the multi-omics datasets into a single two-dimensional matrix. However, unfolding the data and analyzing them using two-way methods may lead to information loss since it breaks the modular structure inherent in the data. Therefore, a tensor data fusion approach will allow us to arrange the data from a set of multi-omics datasets as one single data structure $\mathcal {T}$ called a tensor. This tensor $\mathcal {T}$ has data in a form of a three-way tensor $\mathcal {X} \in \mathbb {R}^{ A \times B \times C}$ where *A* represents the number of multi-omics datasets, *B* represents the number of features in each omic dataset, and *C* is the total number of patients. The structure of this tensor is shown in Fig. 22

.

#### Multi-omics clustering and prediction models

In this section, the patients are stratified into low and high-risk groups using the latent features from the integrated multi-omics data: SCNV, miRNA, RNAseq, and methylation. To rationally identify different subsets of patients associated with different overall survival (OS), a hierarchical clustering with the combination of Canberra distance and ward linkage was used. The Canberra distance serves as a measure of dissimilarity between data points. Meanwhile, Ward’s linkage method determines how clusters are merged by minimizing the total within-cluster variance, leading to the formation of more compact clusters. By combining the Canberra distance for dissimilarity measurement and Ward linkage for cluster merging, the hierarchical clustering algorithm iteratively constructs a dendrogram, providing insights into the hierarchical structure of the data and facilitating the identification of meaningful clusters based on their similarities.

To demonstrate a different performance of low and high-risk groups, prognostic significance is evaluated using univariate (Kaplan-Meier) and multivariate (Cox-regression) models across treated patients from the Breast and Glioma datasets. The *p*-value evaluates the statistically significant level. Further, the tumor purity classification model is developed on the Breast data as it is available only in the clinical Breast data. Tumor purity is an important medical feature that explains the proportion of cancer cells^31,32^. We categorize the tumor purity level into high and low levels based on the threshold of 0.7. The patient is considered a high purity level when the tumor purity value is greater than or equal to 0.7 and low otherwise^33^. It is worth mentioning that the data is divided into the training and testing sets for the clustering and classification models.

### Shapley additive explanation (SHAP)

SHAP (SHapley Additive exPlanations) is a popular technique in explainable artificial intelligence for explaining the output of machine learning models by attributing the importance of each feature to the model’s prediction^34^. It is a model-agnostic approach based on the SHAP values, which provide insights into how each feature contributes to the model’s predictions for individual data points. In this study, we used Kernal and Gradient SHAP method.

#### Kernel SHAP method

The Kernel SHAP method is employed for methylation, miRNA, and SCNV to elucidate the significant biomarkers contributing to the latent variables of the autoencoder^35^. SHAP values interpret the autoencoder specifically in the context of explaining the contribution of each feature to the latent variable. Kernel SHAP applies a specially weighted local linear regression, where the SHAP kernel determines the weights to approximate the SHAP values. It observes how the latent variables change when the feature is included versus excluded from the model.

Autoencoder compress input data into a lower-dimensional latent space and then reconstructs the input from this compressed form. Let $x \in \mathbb {R}^d$ represent the input features, and let $z \in \mathbb {R}^k$ denote the latent variables, where $k < d$. The encoding function can be denoted as $z = g(x)$, and the decoding function as $\hat{x} = h(z)$. To quantify the impact of each input feature on the latent variables using the Kernel SHAP method, we consider the change in the encoded representation as

9 $$\begin{aligned} \phi _i^z = \sum _{S \subseteq \{1, \ldots , d\} \setminus \{i\}} \frac{|S|!(d - |S| - 1)!}{d!} [g_x(S \cup \{i\}) - g_x(S)] \end{aligned}$$

where $\phi _i^z$ represents the SHAP value for feature *i* with respect to the latent variable *z*, and $g_x(S)$ represents the encoder output when only the features in set *S* are active.

#### Gradient SHAP method

For RNAseq, we have used SHAP Gradient Explainer, a variant of SHAP tailored specifically for interpreting predictions from differentiable models, such as those built using deep learning frameworks with large features. In our case, it leverages the idea that gradients-how much a change in an input feature changes the latent variable-can provide insights into the model’s decision-making process. By integrating these gradients, the method can quantify the sensitivity of the latent space to each input feature. The SHAP value for feature *i* using the Gradient SHAP method can be approximated by

10 $$\begin{aligned} \phi _i^z \approx (x_i - x'_i) \times \left. \frac{\partial g(x)}{\partial x_i} \right| _{x=x'} \end{aligned}$$

where $\phi _i^z$ is the approximate SHAP value for feature *i* affecting the latent variable *z*, and $\frac{\partial g(x)}{\partial x_i}$ is the gradient of the encoder output to feature *i*, evaluated at a baseline input $x'$.

### Gene ontology (GO) enrichment analysis

We have used GO enrichment analysis to understand the effect of important genes that act as a biomarker on a latent variable. This identifies which biological processes, cellular components, or molecular functions are overrepresented. The analysis compares the frequency of each GO term in our gene set obtained using SHAP values against a background set, usually representing the entire genome or a relevant subset. For this, we have used PANTHER classification system^36^, which is regularly updated with GO annotations. The outcome of the GO enrichment analysis helps identify the biological significance of the latent variables derived from the autoencoder model. The *p*-value in analysis measures the statistical significance of the overrepresentation of GO terms in a set of genes of interest compared to a background set, whereas fold enrichment is a measure that helps to understand the magnitude of overrepresentation of a particular feature, such as a GO term, within a subset of interest compared to a background set. It can be defined as,

11 $${\text {Fold Enrichment}} = \frac{\text {{Proportion of genes with the GO term in the subset}}}{\text {{Proportion of genes with the GO term in the background}}}$$

## Conclusion

We propose a multi-omics framework utilizing deep-learning autoencoders and tensors to identify cancer risk groups. Multi-omics integrates diverse data types, including methylation, somatic copy-number variation (SCNV), microRNA (miRNA), and RNA sequencing (RNAseq). Our framework employs autoencoders for each omics dataset separately to reduce dimensions and capture maximum information. Latent variables are extracted from individual omics data and integrated using tensors, followed by identification of common features using CANDECOMP/PARAFAC (CP) decomposition. The low-dimensional multi-omics data is clustered into two and three risk groups using hierarchical clustering. Several survival analysis experiments indicate that low-dimensional multi-omics data can be stratified into high and low-risk groups. Furthermore, we employed SHAP to identify the biomarker’s impact. Also, a classification model is constructed using fused features from multi-omics data to predict tumor purity in Breast cancer. Future directions will incorporate biological knowledge to further investigate the interrelationships among different techniques and molecules.

## Data Availability

All the data used in this paper is in the public domain, as stated in the main text. All the original data used in this study are freely available at the Firehose of the Broad Institute (http://gdac.broadinstitute.org/). The Glioma dataset can be downloaded from https://gdac.broadinstitute.org/runs/stddata__2016_01_28/data/GBMLGG/20160128/, and the Breast Invasive Carcinoma dataset can be found at https://gdac.broadinstitute.org/runs/stddata__2016_01_28/data/BRCA/20160128/.

## Code availability

The software package (in Python)and datasets are available from the GitHub link https://github.com/alibraytee/AE-Tensor-Genomics.
